# Supplementary material for: Multi-task weak supervision enables anatomically-resolved abnormality detection in whole-body FDG-PET/CT
Source: Nat Commun. 2021 Mar 25;12:1880. doi: 10.1038/s41467-021-22018-1 (PMC7994797; doi:10.1038/s41467-021-22018-1)
Supplement: Supplementary file 1 — Supplementary Information [file 41467_2021_22018_MOESM1_ESM.pdf]

Supplementary materials for

Multi-task weak supervision enables anatomically-resolved abnormality detection in whole-body FDG-PET/CT

Sabri Eyuboglu[*‡1], Geoffrey Angus[*1], Bhavik Patel[2], Anuj Pareek[2], Guido Davidzon[2], Jin Long[3], Jared Dunnmon,[**1] Matthew P. Lungren [**2]

[1] Department of Computer Science, Stanford University, Stanford, CA 94305, USA

[2] Department of Radiology, Stanford University, Stanford, CA 94305, USA

[3] Center for Artificial Intelligence in Medicine and Imaging, Stanford University, Stanford, CA 94305, USA

[*] [**] Equal contribution; ‡Corresponding author, Email: eyuboglu@stanford.edu

This PDF file includes:

Supplementary Notes 1 to 4

Supplementary Figures 1 to 4

Supplementary Tables 1 to 20

Supplementary References

In this document, we provide additional details on our dataset and methods, several auxiliary analyses of model performance, visualizations of our data, and supplementary tables with detailed results.

Supplementary Note 1 Dataset

We began with a raw dataset of 18,497 PET-CT exams across 11,251 patients, extracted from Stanford Hospital's archival records. We omitted all exams for which the PET and CT channels were fused. Of those that had separate PET and CT channels, we filtered out those exams with fewer than 50 slices, those with a length disparity between the two modalities greater than 2 slices, and those taken using head PET-CT protocols. The resulting dataset consisted of 8,144 exams from 4,691 patients.

Each scan in our dataset ran from the upper thigh to the base of skull and consisted of separate PET and CT channels. Each slice of the scan was stored in an individual DICOM file and both the exam metadata and imaging data were extracted using the `pydicom` package. If the number of PET and CT slices differed, the larger of the two was truncated.

All of the DICOM files were anonymized prior to this study. The exams in our dataset were conducted from 2003 to 2010, using GE Discovery LS and GE Discovery QX/i scanners. We stored the metadata and images in HDF5 format for training.

Supplementary Note 2 Scan pre-processing

We preprocess each FDG-PET/CT scan by: (1) upscaling 128×128 resolution PET images and downscaling 512×512 resolution CT images to a common resolution of 224×224 pixels, (2) normalizing each sequence so that its pixels have a mean value of 0 and a standard deviation of 1, (3) stacking the now equally-sized PET and CT image sequences to create a two channel image sequence. The PET images were upsampled using bilinear interpolation as defined by the Python implementation of OpenCV.

Supplementary Note 3 Model Training

All of our models are trained using `PyTorch v1.0` [1]. During training, only one PET/CT exam at a time can fit on a 12GB GPU. We train all of our models on two Nvidia Titan Xp GPUs with a batch size of two. We tuned the hyperparameters of a single-task abnormality detection model pre-trained on Kinetics using summary code labels. We performed a search over learning rate and dropout probability, finding the highest validation AUROC with a learning rate of 0.0001 and a dropout probability of 0. We use these hyperparameters for all subsequent experiments.

Our report model uses the architecture of Devlin *et al.* [2] and implementation of Wolf *et al.* [3]. We use pretrained weights from Wolf *et al.* [3]. We train all of our models on two Nvidia Titan Xp GPUs with a batch size of 16.

Supplementary Note 4 Regular Expression Baseline

We compare our proposed labeling framework to a rule-based baseline. We describe the procedure used by the baseline in this section. First, the baseline runs the same tagging functions as our labeling framework. As we discuss in *Methods*, each region in our ontology is accompanied by a set of tagging functions that search the report for mentions of the region. For each mention of a region, if there are no terms suggestive of a neutral or negative finding, then the baseline labels the region as abnormal. After running through all of the tagging functions, if the region has not yet been labeled, the region as labeled normal. Finally, we propagate labels up the regional ontology using the same approach as our proposed labeling framework (see *Methods*. We outline the procedure in Algorithm 1, below.

Algorithm 1: Ruled-based Labeler

```
for report in dataset do
    labels = {};
    remove section headers from report;
    for region in ontology do
        for tagging function associated with region do
            if tagging function finds mention of region and
              sentence does not include negative terms then
                labels[region] = 1;
                break;
            end
        end
        if region is not in labels then
            labels[region] = 0;
        end
    end
end
```

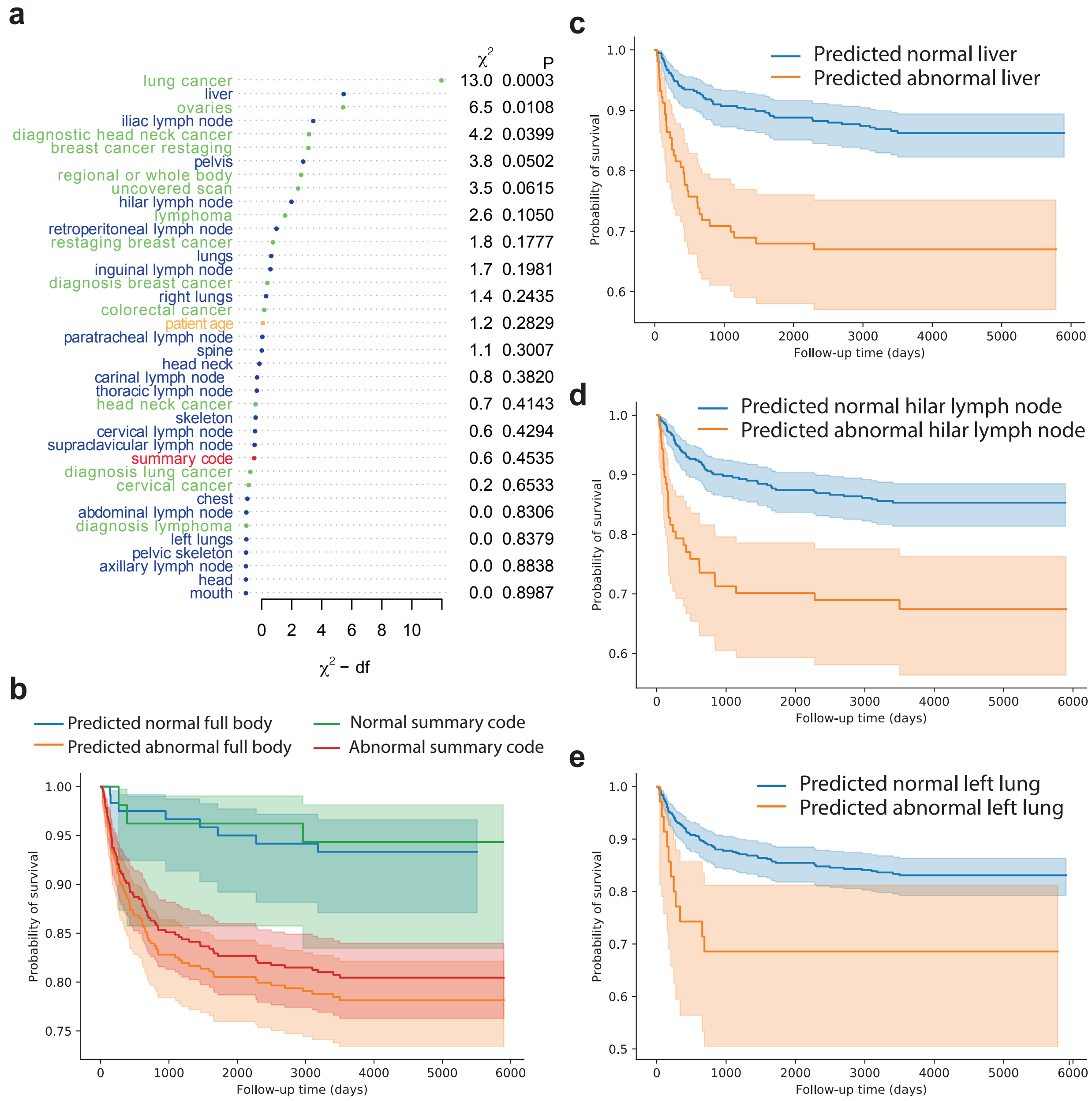

Supplementary Figure 1: (a) Importance of covariates in a multivariable Cox proportional hazard model as measured by Wald chi-square minus the predictor degree of freedom ($\chi^2 - \mathrm{df}$) **(b)** Kaplan-Meier curves showing survival stratified by full body abnormality predictions. To convert the probabilities output by the PET/CT model into full body abnormality predictions we use a threshold of 0.8. Because of severe class imbalance in the dataset, a threshold of 0.5 yields no normal predictions. Also shown are Kaplan-Meier curves stratified by summary code. Error bands show exponential greenwood confidence intervals (95%). **(c-e)** Kaplan-Meier curves showing survival stratified by PET/CT abnormality localization predictions in the (c) liver, (d) hilar lymph node, and (e) left lung. To convert the probabilities output by the PET/CT model to binary predictions by which we can stratify we use a threshold of 0.5. Error bands show exponential greenwood confidence intervals (95%).

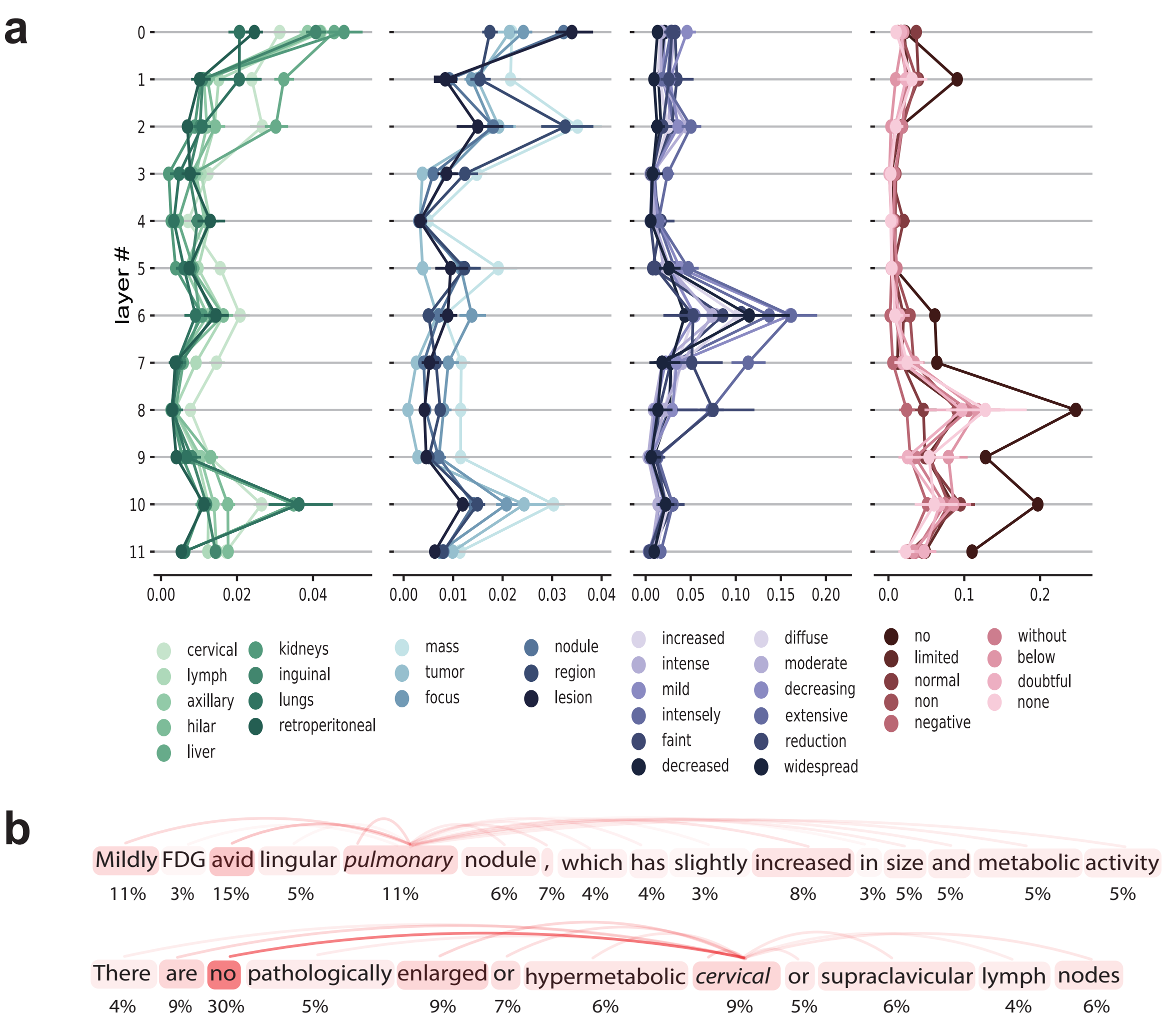

Supplementary Figure 2: Visualizations of our report model's attention distribution. **(a)** The mean self-attention score across all twelve Transformer layers for a sample of clinically relevant words. The words are placed into semantic groups (from left to right): anatomical regions (e.g. lungs, kidneys), features of interest (e.g. mass, lesion), modifiers describing FDG uptake (e.g. mild, widespread), and words of negation (e.g. no, without). We find that the attention patterns across layers are consistent within each semantic group: for example, the modifiers (middle right) are often attended to in the seventh layer, and that the words of negation (far right) are attended to in the final layers. **(b)** The self-attention scores assigned by the mention of the anatomical region (e.g. "pulmonary") to the other tokens in the sentence. The scores are averaged across all twelve layers of the Transformer.

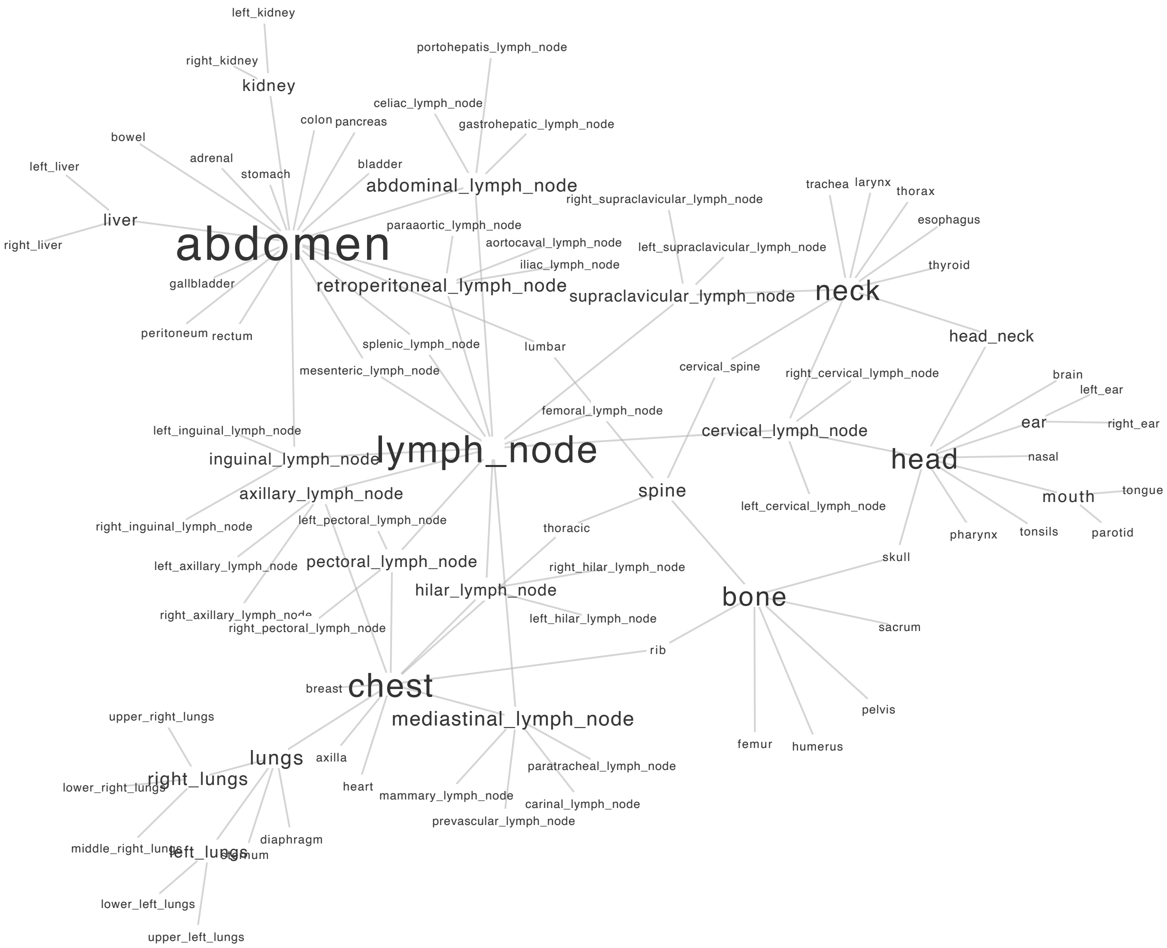

Supplementary Figure 3: Our regional ontology. Each word in this directed acyclic graph represents one of 96 regions in the body. An edge between two regions represents a parent-child relation (e.g. abdomen $\rightarrow$ stomach). The regions included are those most commonly mentioned in our dataset (frequency computed via a k-gram histogram analysis). The graph topology was constructed in consultation with nuclear medicine specialists.

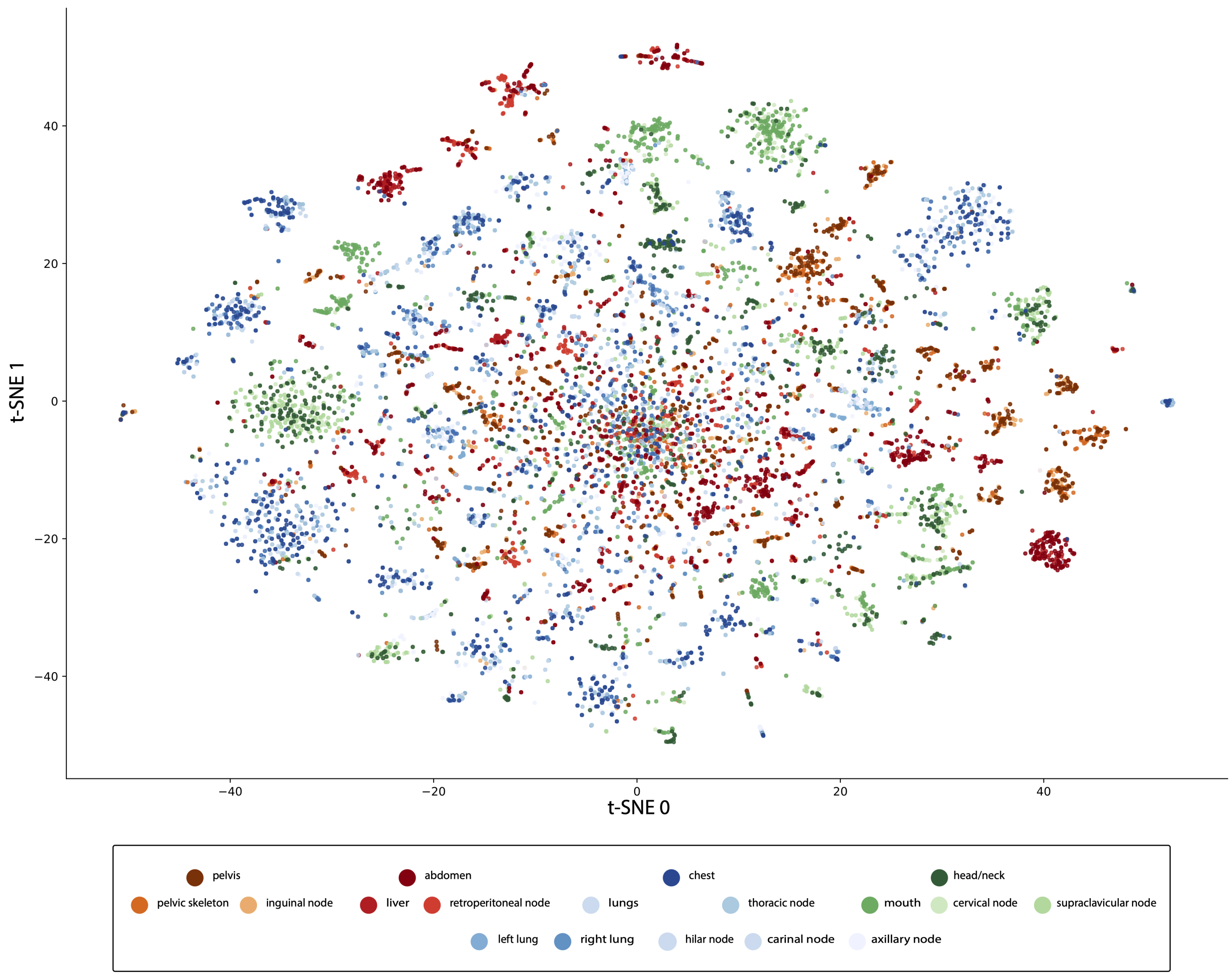

Supplementary Figure 4: A t-SNE projection of model activations prior to the final classification layer ($\mathbf{a} \in \mathbb{R}^d$, see *Methods*). Each point in the plot represents the activation of one of 26 task heads for one of the 800 exams in our test set. Each task head is represented by a different color. Tasks heads that are in the same sub-tree of the ontology share similar colors. We find that the activations cluster according to proximity in the regional ontology.

	**Train** (generated)		**Validation** (generated)		**Test** (hand labeled)	
**Region**	Normal	Abnormal	Normal	Abnormal	Normal	Abnormal
Abdomen	4093	2437	459	341	592	222
Abdominal Lymph Node	5665	865	682	118	756	58
Axillary Lymph Node	5849	681	721	79	734	80
Carinal Lymph Node	5938	592	735	65	769	45
Cervical Lymph Node	5925	605	742	58	709	105
Chest	2997	3533	381	419	456	358
Full	1000	5530	69	731	200	614
Head	4685	1845	599	201	696	118
Head Neck	3385	3145	440	360	532	282
Hilar Lymph Node	5348	1182	680	120	713	101
Iliac Lymph Node	5796	734	720	80	767	47
Inguinal Lymph Node	5941	589	746	54	758	56
Left Lungs	5967	563	719	81	715	99
Liver	5705	825	685	115	745	69
Lungs	4905	1625	590	210	630	184
Mouth	5747	783	727	73	751	63
Neck	4415	2115	543	257	602	212
Paratracheal Lymph Node	5920	610	725	75	758	56
Pelvic Skeleton	5863	667	727	73	755	59
Pelvis	5073	1457	636	164	635	179
Retroperitoneal Lymph Node	5745	785	718	82	743	71
Right Lungs	5927	603	709	91	703	111
Skeleton	3976	2554	505	295	675	139
Spine	5089	1441	638	162	749	65
Supraclavicular Lymph Node	5799	731	721	79	747	67
Thoracic Lymph Node	4468	2062	576	224	651	163
(Rare) Adrenal	6342	188	766	34	797	17
(Rare) Celiac Lymph Node	6456	74	793	7	796	18
(Rare) Kidney	6336	194	781	19	810	4
(Rare) Pancreas	6329	201	769	31	804	10

Supplementary Table 1: The class counts for each of the 26 regions used to train the multi-task model, plus the 4 additional rare tasks. The training and validation set columns show the labels as predicted by our labeling framework with threshold 0.5). The test set columns show the labels as assigned via manual annotation.

Task	Multi-task (with fine-tuning)	Single-task (Kinetics pretraining)
Abdomen	**0.794 (0.788, 0.802)**	0.771 (0.754, 0.789)
Abdominal Lymph Node	**0.815 (0.800, 0.832)**	0.801 (0.792, 0.808)
Axillary Lymph Node	**0.914 (0.911, 0.919)**	0.892 (0.878, 0.906)
Carinal Lymph Node	**0.903 (0.897, 0.911)**	0.884 (0.870, 0.898)
Cervical Lymph Node	**0.772 (0.762, 0.781)**	0.675 (0.592, 0.781)
Chest	0.848 (0.841, 0.855)	**0.870 (0.861, 0.879)**
Full	**0.803 (0.782, 0.823)**	0.751 (0.721, 0.789)
Head	**0.709 (0.700, 0.718)**	0.704 (0.667, 0.752)
Head Neck	**0.783 (0.774, 0.792)**	0.753 (0.736, 0.774)
Hilar Lymph Node	**0.908 (0.904, 0.911)**	0.904 (0.887, 0.923)
Iliac Lymph Node	**0.773 (0.769, 0.777)**	0.675 (0.644, 0.715)
Inguinal Lymph Node	0.894 (0.889, 0.898)	**0.900 (0.896, 0.905)**
Left Lungs	**0.854 (0.845, 0.863)**	0.759 (0.672, 0.895)
Liver	**0.905 (0.895, 0.915)**	0.881 (0.876, 0.887)
Lungs	0.860 (0.851, 0.868)	**0.905 (0.903, 0.908)**
Mouth	**0.751 (0.738, 0.763)**	0.742 (0.720, 0.772)
Neck	0.775 (0.763, 0.783)	**0.798 (0.791, 0.807)**
Paratracheal Lymph Node	**0.860 (0.851, 0.871)**	0.813 (0.799, 0.829)
Pelvic Skeleton	0.692 (0.669, 0.708)	**0.710 (0.691, 0.736)**
Pelvis	**0.788 (0.784, 0.792)**	0.785 (0.774, 0.796)
Retroperitoneal Lymph	0.816 (0.793, 0.838)	**0.826 (0.817, 0.836)**
Right Lungs	0.823 (0.815, 0.831)	**0.826 (0.811, 0.841)**
Skeleton	0.684 (0.668, 0.700)	**0.719 (0.702, 0.732)**
Spine	0.665 (0.633, 0.685)	**0.742 (0.723, 0.759)**
Supraclavicular Lymph	**0.867 (0.859, 0.876)**	0.852 (0.836, 0.868)
Thoracic Lymph Node	0.869 (0.863, 0.877)	**0.881 (0.876, 0.888)**

Supplementary Table 2: The mean AUROC of our fine-tuned, multi-task models and the single-task models on each of the 26 regions (95% confidence intervals computed via bootstrapping).

Task	Fully supervised	Weakly supervised	Fully + weakly supervised
Abdomen	0.506 (0.478, 0.531)	**0.820 (0.806, 0.834)**	0.819 (0.806, 0.831)
Abdominal lymph node	0.476 (0.434, 0.524)	**0.777 (0.764, 0.789)**	0.745 (0.731, 0.759)
Axillary lymph node	0.708 (0.692, 0.724)	**0.930 (0.922, 0.940)**	0.915 (0.907, 0.924)
Carinal lymph node	0.800 (0.790, 0.816)	**0.921 (0.914, 0.925)**	0.919 (0.911, 0.929)
Cervical lymph node	0.427 (0.386, 0.464)	0.795 (0.784, 0.805)	**0.809 (0.802, 0.817)**
Chest	0.773 (0.754, 0.791)	0.832 (0.824, 0.840)	**0.858 (0.855, 0.862)**
Full	0.424 (0.414, 0.432)	0.837 (0.817, 0.855)	**0.850 (0.839, 0.860)**
Head	0.592 (0.575, 0.609)	**0.688 (0.677, 0.703)**	**0.688 (0.683, 0.695)**
Head neck	0.494 (0.481, 0.504)	0.811 (0.802, 0.818)	**0.814 (0.807, 0.821)**
Hilar lymph node	0.841 (0.818, 0.864)	**0.916 (0.910, 0.922)**	0.914 (0.897, 0.931)
Iliac lymph node	0.514 (0.465, 0.553)	0.810 (0.797, 0.823)	**0.830 (0.819, 0.839)**
Inguinal lymph node	0.561 (0.505, 0.612)	0.922 (0.915, 0.930)	**0.935 (0.922, 0.954)**
Left lungs	0.703 (0.684, 0.723)	0.833 (0.818, 0.845)	**0.841 (0.828, 0.856)**
Liver	0.574 (0.524, 0.612)	**0.926 (0.918, 0.934)**	0.924 (0.911, 0.936)
Lungs	0.741 (0.731, 0.750)	0.833 (0.822, 0.842)	**0.874 (0.862, 0.887)**
Mouth	0.568 (0.555, 0.583)	**0.758 (0.734, 0.782)**	0.756 (0.740, 0.771)
Neck	0.435 (0.419, 0.450)	0.792 (0.777, 0.803)	**0.822 (0.814, 0.833)**
Paratracheal lymph node	0.724 (0.714, 0.730)	**0.844 (0.831, 0.859)**	0.825 (0.817, 0.832)
Pelvic skeleton	0.681 (0.652, 0.711)	0.743 (0.717, 0.768)	**0.760 (0.739, 0.781)**
Pelvis	0.569 (0.541, 0.598)	0.813 (0.804, 0.823)	**0.836 (0.821, 0.852)**
Retroperitoneal lymph node	0.400 (0.304, 0.466)	**0.868 (0.841, 0.892)**	0.864 (0.850, 0.881)
Right lungs	0.719 (0.709, 0.727)	0.818 (0.812, 0.826)	**0.851 (0.837, 0.865)**
Skeleton	0.679 (0.660, 0.704)	0.698 (0.677, 0.719)	**0.699 (0.681, 0.722)**
Spine	0.680 (0.660, 0.712)	0.678 (0.645, 0.705)	**0.742 (0.719, 0.766)**
Supraclavicular lymph node	0.321 (0.308, 0.335)	0.873 (0.859, 0.884)	**0.900 (0.893, 0.910)**
Thoracic lymph node	0.784 (0.774, 0.793)	**0.868 (0.864, 0.876)**	0.867 (0.860, 0.875)

Supplementary Table 3: Comparison of supervision strategies for anatomically-resolved abnormality detection. For each supervision strategy and task, we show the mean AUROC over five random seeds of our fine-tuned, multi-task models on each of the 26 regions (95% confidence intervals computed via bootstrapping). Fully supervised models are those trained with a hand-labeled dataset of 400 exams. Weakly supervised models are those trained with a weakly-supervised dataset of 6,530 exams. Weakly + fully supervised models are those trained with a combination of 6,530 weakly-supervised exams and 400 hand-labeled exams.

Task	Baseline	Ours
Abdomen	0.583	**0.697 (0.625, 0.806)**
Abdominal Lymph Node	0.325	**0.425 (0.387, 0.476)**
Axillary Lymph Node	0.693	**0.759 (0.712, 0.814)**
Carinal Lymph Node	**0.615**	0.593 (0.575, 0.610)
Cervical Lymph Node	0.570	**0.624 (0.608, 0.640)**
Chest	0.702	**0.788 (0.756, 0.835)**
Full	0.862	**0.888 (0.878, 0.903)**
Head	0.266	**0.503 (0.421, 0.625)**
Head Neck	0.530	**0.685 (0.631, 0.769)**
Hilar Lymph Node	0.633	**0.702 (0.689, 0.720)**
Iliac Lymph Node	0.485	**0.593 (0.572, 0.621)**
Inguinal Lymph Node	0.598	**0.766 (0.729, 0.817)**
Left Lungs	**0.506**	0.486 (0.473, 0.500)
Liver	0.484	**0.679 (0.634, 0.732)**
Lungs	0.565	**0.701 (0.667, 0.741)**
Mouth	0.440	**0.492 (0.454, 0.553)**
Neck	0.615	**0.685 (0.659, 0.719)**
Paratracheal Lymph Node	0.637	**0.645 (0.628, 0.662)**
Pelvic Skeleton	0.162	**0.243 (0.219, 0.267)**
Pelvis	0.415	**0.680 (0.646, 0.733)**
Retroperitoneal Lymph Node	0.539	**0.686 (0.665, 0.716)**
Right Lungs	0.473	**0.520 (0.501, 0.537)**
Skeleton	0.326	**0.477 (0.429, 0.551)**
Spine	0.309	**0.374 (0.352, 0.401)**
Supraclavicular Lymph Node	0.633	**0.722 (0.688, 0.768)**
Thoracic Lymph Node	0.596	**0.679 (0.648, 0.726)**

Supplementary Table 4: A comparison of a regular expression model baseline and our labeling framework on the task of extracting anatomically-resolved abnormality detection labels from PET/CT reports. Shown is the mean F1 score across five random seeds, (95% confidence interval computed via bootstrapping). Since the baseline is deterministic, changing the random seed has no effect. Bold indicates the performance of the best model for each region.

# of training examples	Full supervision (single-task)	Weak supervision (single-task)	Weak supervision (multi-task)
100	0.524 (0.516, 0.532)	**0.623 (0.602, 0.638)**	0.564 (0.557, 0.571)
1000	0.639 (0.607, 0.679)	**0.685 (0.634, 0.769)**	0.616 (0.520, 0.726)
2000	0.725 (0.704, 0.750)	0.726 (0.701, 0.742)	**0.737 (0.708, 0.772)**
4000	0.756 (0.734, 0.782)	**0.762 (0.737, 0.790)**	0.755 (0.718, 0.802)
6530	0.783 (0.773, 0.793)	0.768 (0.745, 0.790)	**0.803 (0.782, 0.823)**

Supplementary Table 5: Binary whole-body abnormality detection AUROC vs. number of training data points. The columns correspond to different supervision strategies: (1) full supervision using summary codes as labels (in a single-task setting) and (2) weak supervision using labels generated by our labeling framework (in a single-task setting), and (3) weak supervision (in a multi-task abnormality detection setting). Reported is the mean AUROC across five different random seeds (95% confidence interval computed via bootstrapping). Bold indicates the performance of the best model.

Task	Single-task (Kinetics pretraining)	Single-task (summary code pretraining)	Multi-task (with fine-tuning)
45 Days	0.640 (0.530, 0.728)	0.611 (0.544, 0.678)	**0.692 (0.646, 0.725)**
90 Days	0.693 (0.614, 0.758)	0.693 (0.616, 0.767)	**0.799 (0.762, 0.838)**
180 Days	0.591 (0.561, 0.615)	0.641 (0.599, 0.680)	**0.648 (0.584, 0.709)**
365 Days	0.576 (0.542, 0.608)	**0.639 (0.619, 0.656)**	0.607 (0.575, 0.639)

Supplementary Table 6: A comparison of models with various weight initializations on the task of predicting patient mortality within t days, where t is indicated in the first column. Reported is mean AUROC score across five random seeds (95% confidence interval computed via bootstrapping). Bold indicates the best model for each task.

Task	Single-task (Kinetics pretraining)	Single-task (summary code pretraining)	Single-task (in-domain pretraining)	Multi-task (with fine-tuning)
Adrenal	0.580 (0.502, 0.665)	0.582 (0.511, 0.637)	0.604 (0.582, 0.638)	**0.779 (0.762, 0.797)**
Celiac Lymph Node	0.502 (0.435, 0.572)	0.505 (0.405, 0.632)	0.674 (0.647, 0.704)	**0.780 (0.736, 0.849)**
Kidney	0.635 (0.585, 0.690)	0.593 (0.488, 0.698)	0.717 (0.608, 0.783)	**0.899 (0.866, 0.940)**
Pancreas	0.559 (0.510, 0.616)	0.664 (0.607, 0.728)	0.620 (0.574, 0.661)	**0.782 (0.732, 0.833)**

Supplementary Table 7: A comparison of models with various weight initializations on the task of rare abnormality location estimation. Reported is mean AUROC score across five random seeds (95% confidence interval computed via bootstrapping). These tasks are not part of the 26 core tasks used for multi-task pretraining. Bold indicates the performance of the best model for a particular regional task.

Epoch	Single-task (Kinetics pretraining)	Multi-task (with fine-tuning)
1	0.629 (0.613, 0.645)	**0.796 (0.782, 0.810)**
2	0.704 (0.686, 0.722)	**0.807 (0.794, 0.820)**
3	0.747 (0.729, 0.764)	**0.810 (0.798, 0.823)**
4	0.769 (0.752, 0.786)	**0.812 (0.800, 0.825)**
5	0.781 (0.766, 0.797)	**0.813 (0.800, 0.825)**
best	0.801 (0.786, 0.815)	**0.813 (0.800, 0.825)**

Supplementary Table 8: A comparison of training complexity for multi-task and single-task models. We fine-tune our multi-task FDG-PET/CT model in a single-task setting for each of the twenty-six core anatomical region. We do the same with a model pretrained on Kinetics [4], an out-of-domain dataset. Here, we report how mean AUROC across all anatomical regions improves with more epochs of fine-tuning (95% confidence interval computed via bootstrapping). The final row indicates model performance upon convergence. Bold indicates the performance of the best model after training for k epochs.

Task	Sum Reduction	Attention (ours)
Overall	0.759 (0.744, 0.774)	**0.799 (0.785, 0.814)**
Abdomen	0.737 (0.704, 0.793)	**0.778 (0.767, 0.793)**
Abdominal Lymph Node	0.774 (0.716, 0.863)	**0.788 (0.767, 0.807)**
Axillary Lymph Node	0.826 (0.797, 0.873)	**0.879 (0.860, 0.900)**
Carinal Lymph Node	0.851 (0.827, 0.893)	**0.888 (0.881, 0.896)**
Cervical Lymph Node	0.723 (0.691, 0.766)	**0.790 (0.783, 0.797)**
Chest	**0.868 (0.862, 0.874)**	0.822 (0.761, 0.924)
Full	0.797 (0.768, 0.846)	**0.811 (0.790, 0.842)**
Head	0.702 (0.689, 0.715)	**0.727 (0.720, 0.735)**
Head Neck	**0.768 (0.754, 0.785)**	0.662 (0.524, 0.805)
Hilar Lymph Node	0.877 (0.865, 0.889)	**0.908 (0.903, 0.913)**
Iliac Lymph Node	0.670 (0.644, 0.712)	**0.743 (0.728, 0.763)**
Inguinal Lymph Node	0.623 (0.579, 0.666)	**0.865 (0.843, 0.890)**
Left Lungs	0.724 (0.685, 0.766)	**0.824 (0.812, 0.834)**
Liver	0.775 (0.724, 0.847)	**0.855 (0.836, 0.878)**
Lungs	0.859 (0.850, 0.870)	**0.870 (0.856, 0.885)**
Mouth	0.688 (0.654, 0.721)	**0.772 (0.756, 0.788)**
Neck	0.782 (0.771, 0.801)	**0.802 (0.794, 0.811)**
Paratracheal Lymph Node	0.810 (0.777, 0.859)	**0.856 (0.847, 0.864)**
Pelvic Skeleton	0.596 (0.561, 0.631)	**0.668 (0.641, 0.695)**
Pelvis	0.680 (0.657, 0.715)	**0.759 (0.747, 0.774)**
Retroperitoneal Lymph Node	0.785 (0.762, 0.811)	**0.807 (0.786, 0.829)**
Right Lungs	0.749 (0.721, 0.779)	**0.841 (0.831, 0.850)**
Skeleton	**0.680 (0.662, 0.708)**	0.655 (0.615, 0.694)
Spine	**0.707 (0.697, 0.717)**	0.665 (0.603, 0.747)
Supraclavicular Lymph Node	0.807 (0.784, 0.835)	**0.864 (0.843, 0.888)**
Thoracic Lymph Node	0.864 (0.860, 0.869)	**0.875 (0.868, 0.883)**

Supplementary Table 9: An ablation study demonstrating the performance gains enabled by our spatial attention mechanism. Reported is mean AUROC score (95% confidence interval computed via bootstrapping). Bold indicates the performance of the best model for each region.

Task	128 $\times$ 128	224 $\times$ 224 (ours)
Full	0.767 (0.745, 0.787)	**0.803 (0.782, 0.823)**
Inguinal Lymph Node	0.863 (0.843, 0.890)	**0.894 (0.889, 0.898)**
Liver	0.859 (0.849, 0.867)	**0.905 (0.895, 0.915)**
Lungs	0.855 (0.843, 0.863)	**0.860 (0.851, 0.868)**

Supplementary Table 10: An ablation study on a sample of four tasks demonstrating the performance gains enabled by upsampling the PET images to 224 $\times$ 224 pixels.

Task	FDG-PET only	FDG-PET + CT (ours)
Full	0.775 (0.747, 0.803)	**0.803 (0.782, 0.823)**
Inguinal Lymph Node	0.891 (0.879, 0.901)	**0.894 (0.889, 0.898)**
Liver	0.877 (0.870, 0.884)	**0.905 (0.895, 0.915)**
Lungs	0.840 (0.836, 0.845)	**0.860 (0.851, 0.868)**

Supplementary Table 11: An ablation study on a sample of four tasks demonstrating the performance gains enabled by training a model to detect FDG abnormalities using both the FDG-PET and CT modalities.

Age	Test	Train	Validation
0-10	5	42	1
10-20	10	153	29
20-30	42	350	59
30-40	71	474	69
40-50	122	865	102
50-60	193	1401	158
60-70	169	1554	180
70-80	120	1164	135
80-90	81	523	67
Unknown	1	4	0
**Total**	**814**	**6530**	**800**

Supplementary Table 12: Patient Age breakdown of the exams in the dataset.

Study Date	Test	Train	Validation
2003	8	64	13
2004	17	110	11
2005	27	139	19
2006	98	968	118
2007	165	1310	175
2008	180	1573	175
2009	181	1297	172
2010	138	1069	117
**Total**	**814**	**6530**	**800**

Supplementary Table 13: Study date breakdown of the exams in the dataset.

Scanner Type	Test	Train	Validation
GEMS Discovery LS	801	6433	785
GEMS Discovery QX/i	13	97	15
**Total**	**814**	**6530**	**800**

Supplementary Table 14: Scanner type breakdown of the exams in the dataset.

Cancer Type	Test	Train	Validation
Brain Cancer	2	17	3
Breast Cancer	36	298	36
Cervical Cancer	21	105	6
Colorectal Cancer	41	367	43
Esophageal Cancer	11	120	11
Head/Neck Cancer	122	821	68
Lung Cancer	91	775	111
Lymphoma	263	2415	297
Melanoma	28	193	32
Myeloma	1	22	2
Ovarian Cancer	6	16	2
Pancreatic Cancer	0	25	5
Sarcoma	2	18	2
Testicular Cancer	0	4	0
Thyroid Cancer	0	27	6
Other	190	1307	176
Total	814	6530	800

Supplementary Table 15: Cancer type breakdown of the exams in the dataset. The cancer type is derived from the Study Description entry in the DICOM headers of the exam.

**Covariate**	**Single Variable** log HR (95% CI)	p	**Multivariable** log HR (95% CI)	p
patient age	0.007 (-0.007, 0.020)	0.3120	-0.008 (-0.022, 0.006)	0.2829
summary code	1.318 (0.165, 2.470)	0.0250	0.463 (-0.748, 1.675)	0.4535
abnormality: carinal lymph node	0.857 (0.218, 1.496)	0.0086	-0.710 (-2.301, 0.881)	0.3820
abnormality: inguinal lymph node	0.722 (-0.035, 1.480)	0.0614	-1.009 (-2.546, 0.528)	0.1981
abnormality: left lungs	1.449 (0.757, 2.141)	$<$1e-4	-0.162 (-1.711, 1.388)	0.8379
abnormality: cervical lymph node	0.783 (-0.946, 2.511)	0.3748	1.282 (-1.898, 4.461)	0.4294
abnormality: skeleton	1.547 (0.568, 2.526)	0.0019	-0.790 (-2.695, 1.115)	0.4164
abnormality: chest	1.369 (0.744, 1.994)	$<$1e-4	-0.216 (-1.459, 1.026)	0.7331
abnormality: head neck	0.721 (0.048, 1.394)	0.0358	0.649 (-0.676, 1.974)	0.3372
abnormality: thoracic lymph node	0.937 (0.380, 1.494)	0.0010	-0.804 (-2.637, 1.028)	0.3898
abnormality: lungs	1.455 (0.793, 2.117)	$<$1e-4	1.346 (-0.678, 3.371)	0.1925
abnormality: hilar lymph node	1.152 (0.600, 1.704)	$<$1e-4	1.499 (-0.184, 3.183)	0.0808
abnormality: pelvis	1.329 (0.747, 1.911)	$<$1e-4	1.208 (-0.001, 2.418)	0.0502
abnormality: spine	0.661 (-0.382, 1.705)	0.2143	0.925 (-0.827, 2.678)	0.3007
abnormality: head	0.892 (0.150, 1.634)	0.0184	-0.141 (-2.194, 1.912)	0.8930
abnormality: liver	1.448 (0.903, 1.993)	$<$1e-4	1.316 (0.307, 2.325)	0.0106
abnormality: supraclavicular lymph node	1.410 (0.154, 2.666)	0.0278	-0.967 (-3.429, 1.494)	0.4412
abnormality: retroperitoneal lymph node	0.592 (-0.443, 1.626)	0.2623	-1.340 (-3.174, 0.495)	0.1523
abnormality: abdominal lymph node	1.379 (0.485, 2.272)	0.0025	-0.237 (-2.405, 1.932)	0.8306
abnormality: mouth	0.803 (-0.091, 1.697)	0.0784	-0.141 (-2.312, 2.030)	0.8987
abnormality: axillary lymph node	0.704 (0.009, 1.399)	0.0471	0.091 (-1.130, 1.312)	0.8838
abnormality: paratracheal lymph node	1.060 (0.291, 1.829)	0.0069	0.992 (-0.847, 2.831)	0.2903
abnormality: right lungs	1.316 (0.328, 2.304)	0.0090	-1.216 (-3.260, 0.827)	0.2435
abnormality: pelvic skeleton	2.169 (1.277, 3.061)	$<$1e-4	0.180 (-1.886, 2.247)	0.8641
abnormality: iliac lymph node	1.752 (1.012, 2.492)	$<$1e-4	1.770 (0.136, 3.405)	0.0338
indication: breast cancer restaging	0.534 (-0.470, 1.539)	0.2969	1.457 (0.060, 2.854)	0.0409
indication: cervical cancer	-0.694 (-2.665, 1.278)	0.4903	0.511 (-1.719, 2.741)	0.6533
indication: colorectal cancer	-0.071 (-1.075, 0.933)	0.8898	0.816 (-0.619, 2.252)	0.2651
indication: head neck cancer	-0.595 (-1.368, 0.179)	0.1321	0.526 (-0.738, 1.790)	0.4143
indication: lung cancer	1.060 (0.501, 1.619)	0.0002	2.166 (0.990, 3.341)	0.0003
indication: lymphoma	-0.405 (-0.997, 0.186)	0.1791	0.923 (-0.193, 2.039)	0.1050
indication: ovaries	0.835 (-0.568, 2.239)	0.2432	2.367 (0.547, 4.187)	0.0108
indication: uncovered scan misc	0.230 (-0.430, 0.891)	0.4944	1.087 (-0.052, 2.226)	0.0615
indication: diagnosis breast cancer	0.608 (-1.363, 2.580)	0.5455	1.405 (-0.886, 3.697)	0.2293
indication: diagnosis lung cancer	-0.072 (-1.474, 1.331)	0.9201	0.501 (-1.261, 2.262)	0.5776
indication: diagnosis lymphoma	-1.026 (-2.429, 0.377)	0.1516	0.182 (-1.536, 1.899)	0.8358
indication: diagnostic head neck cancer	0.553 (-0.600, 1.705)	0.3473	1.622 (0.075, 3.168)	0.0399
indication: regional or whole body	0.355 (-0.219, 0.929)	0.2254	1.109 (-0.022, 2.241)	0.0545
indication: restaging breast cancer	0.969 (-1.003, 2.941)	0.3353	1.585 (-0.720, 3.889)	0.1777

Supplementary Table 16: Log hazard ratios for single variable Cox models fit on each covariate and a multivariable Cox model fit on all covariates jointly. Shown are Wald 95% confidence intervals and p values.

**Covariate**	log HR (95% CI)	p
patient age	0.001 (-0.013, 0.015)	0.8669
summary code	1.167 (-0.004, 2.337)	0.0507
indication: breast cancer restaging	1.257 (-0.060, 2.574)	0.0614
indication: cervical cancer	0.179 (-1.978, 2.337)	0.8705
indication: colorectal cancer	0.584 (-0.731, 1.900)	0.3839
indication: head neck cancer	0.188 (-0.961, 1.338)	0.7480
indication: lung cancer	1.572 (0.556, 2.588)	0.0024
indication: lymphoma	0.416 (-0.623, 1.456)	0.4324
indication: ovaries	1.449 (-0.194, 3.093)	0.0839
indication: uncovered scan misc	0.864 (-0.213, 1.940)	0.1158
indication: diagnosis breast ca	1.237 (-0.919, 3.393)	0.2607
indication: diagnosis lung cancer	0.612 (-1.028, 2.252)	0.4647
indication: diagnosis lymphoma	-0.158 (-1.807, 1.492)	0.8514
indication: diagnostic head neck ca	1.232 (-0.201, 2.665)	0.0919
indication: regional or whole body	0.995 (-0.029, 2.019)	0.0569
indication: restaging breast ca	2.025 (-0.137, 4.188)	0.0665

Supplementary Table 17: Log hazard ratios for a multivariable Cox model fit on indication, patient age and summary code. Shown are Wald 95% confidence intervals and p values.

**Covariate**	log HR (95% CI)	p
abnormality: carinal lymph node	-0.027 (-1.514, 1.460)	0.9717
abnormality: inguinal lymph node	-1.223 (-2.523, 0.076)	0.0650
abnormality: left lungs	-0.079 (-1.468, 1.309)	0.9107
abnormality: cervical lymph node	0.923 (-2.286, 4.131)	0.5730
abnormality: skeleton	-0.871 (-2.699, 0.956)	0.3502
abnormality: chest	0.119 (-1.060, 1.298)	0.8432
abnormality: head neck	0.284 (-0.992, 1.561)	0.6623
abnormality: thoracic lymph node	-0.440 (-2.141, 1.261)	0.6120
abnormality: lungs	1.645 (-0.261, 3.551)	0.0907
abnormality: hilar lymph node	0.707 (-0.852, 2.266)	0.3742
abnormality: pelvis	1.180 (0.040, 2.320)	0.0425
abnormality: spine	0.612 (-1.172, 2.395)	0.5016
abnormality: head	-0.194 (-1.896, 1.508)	0.8233
abnormality: liver	1.379 (0.465, 2.293)	0.0031
abnormality: supraclavicular lymph node	-0.579 (-2.960, 1.802)	0.6338
abnormality: retroperitoneal lymph node	-0.853 (-2.567, 0.862)	0.3298
abnormality: abdominal lymph node	-0.692 (-2.763, 1.379)	0.5127
abnormality: mouth	0.148 (-1.726, 2.022)	0.8770
abnormality: axillary lymph node	0.159 (-0.926, 1.243)	0.7742
abnormality: paratracheal lymph node	0.683 (-1.122, 2.487)	0.4585
abnormality: right lungs	-1.436 (-3.428, 0.556)	0.1577
abnormality: pelvic skeleton	0.046 (-1.888, 1.980)	0.9627
abnormality: iliac lymph node	1.586 (0.016, 3.157)	0.0478

Supplementary Table 18: Log hazard ratios for a multivariable Cox model fit on abnormality location predictions. Shown are Wald 95% confidence intervals and p values.

Task	without predictions	with predictions
45 Days	0.300	**0.832**
90 Days	0.613	**0.844**
180 Days	0.601	**0.765**
365 Days	0.558	**0.706**

Supplementary Table 19: Comparison of logistic regression models trained to predict x-day mortality with and without abnormality location predictions included in the covariates. Other covariates used in both models are age, indication, exam summary code (see Mortality Prediction and Survival Analysis in Methods). Logistic regression models are fit on the validation set. The table shows AUROC computed on the test set.

Task	Weak Labels (multi-task)	Ensemble
Abdomen	0.794 (0.788, 0.802)	**0.808**
Abdominal Lymph Node	0.815 (0.800, 0.832)	**0.834**
Axillary Lymph Node	0.914 (0.911, 0.919)	**0.924**
Carinal Lymph Node	0.903 (0.897, 0.911)	**0.912**
Cervical Lymph Node	0.772 (0.762, 0.781)	**0.792**
Chest	0.848 (0.841, 0.855)	**0.870**
Full	0.803 (0.782, 0.823)	**0.825**
Head	0.709 (0.700, 0.718)	**0.719**
Head Neck	0.783 (0.774, 0.792)	**0.801**
Hilar Lymph Node	0.908 (0.904, 0.911)	**0.920**
Iliac Lymph Node	0.773 (0.769, 0.777)	**0.788**
Inguinal Lymph Node	0.894 (0.889, 0.898)	**0.900**
Left Lungs	0.854 (0.845, 0.863)	**0.875**
Liver	0.905 (0.895, 0.915)	**0.911**
Lungs	0.860 (0.851, 0.868)	**0.879**
Mouth	0.751 (0.738, 0.763)	**0.763**
Neck	0.775 (0.763, 0.783)	**0.788**
Paratracheal Lymph Node	0.860 (0.851, 0.871)	**0.876**
Pelvic Skeleton	0.692 (0.669, 0.708)	**0.722**
Pelvis	0.788 (0.784, 0.792)	**0.799**
Retroperitoneal Lymph	0.816 (0.793, 0.838)	**0.839**
Right Lungs	0.823 (0.815, 0.831)	**0.853**
Skeleton	0.684 (0.668, 0.700)	**0.702**
Spine	0.665 (0.633, 0.685)	**0.690**
Supraclavicular Lymph	0.867 (0.859, 0.876)	**0.884**
Thoracic Lymph Node	0.869 (0.863, 0.877)	**0.884**

Supplementary Table 20: The performance of our weakly supervised, multi-task model on each of the 26 tasks, compared to an ensemble model. The ensemble model averages the predictions made by each of the 5 models with differing random seed initializations.

Supplementary References

1. Paszke, A. *et al.* Pytorch: An imperative style, high-performance deep learning library. In Wallach, H. *et al.* (eds.) *Advances in Neural Information Processing Systems 32*, 8024–8035 (Curran Associates, Inc., 2019).

2. Devlin, J., Chang, M.-W., Lee, K. & Toutanova, K. BERT: Pre-training of Deep Bidirectional Transformers for Language Understanding. *arXiv:1810.04805 [cs]* (2018). ArXiv: 1810.04805.

3. Wolf, T. *et al.* Huggingface's transformers: State-of-the-art natural language processing. *ArXiv* **abs/1910.03771** (2019).

4. Carreira, J., Zisserman, A., Com, Z. & Deepmind, Quo Vadis, Action Recognition? A New Model and the Kinetics Dataset. Tech. Rep.
