# Supplementary material for: Multi-task weak supervision enables anatomically-resolved abnormality detection in whole-body FDG-PET/CT
Source: Nat Commun. 2021 Mar 25;12:1880. doi: 10.1038/s41467-021-22018-1 (PMC7994797; doi:10.1038/s41467-021-22018-1)
Supplement: Supplementary file 3 — Reporting Summary [file 41467_2021_22018_MOESM3_ESM.pdf]

nature research

Corresponding author(s): Sabri Eyuboglu

Last updated by author(s): March 29, 2020

Reporting Summary

Nature Research wishes to improve the reproducibility of the work that we publish. This form provides structure for consistency and transparency in reporting. For further information on Nature Research policies, seeAuthors & Referees and theEditorial Policy Checklist .

Statistics

For all statistical analyses, confirm that the following items are present in the figure legend, table legend, main text, or Methods section.

n/a	Confirmed	
☐	☒	The exact sample size (*n*) for each experimental group/condition, given as a discrete number and unit of measurement
☐	☒	A statement on whether measurements were taken from distinct samples or whether the same sample was measured repeatedly
☐	☒	The statistical test(s) used AND whether they are one- or two-sided<br>*Only common tests should be described solely by name; describe more complex techniques in the Methods section.*
☐	☒	A description of all covariates tested
☐	☒	A description of any assumptions or corrections, such as tests of normality and adjustment for multiple comparisons
☐	☒	A full description of the statistical parameters including central tendency (e.g. means) or other basic estimates (e.g. regression coefficient) AND variation (e.g. standard deviation) or associated estimates of uncertainty (e.g. confidence intervals)
☐	☒	For null hypothesis testing, the test statistic (e.g. *F*, *t*, *r*) with confidence intervals, effect sizes, degrees of freedom and *P* value noted<br>*Give P values as exact values whenever suitable.*
☒	☐	For Bayesian analysis, information on the choice of priors and Markov chain Monte Carlo settings
☒	☐	For hierarchical and complex designs, identification of the appropriate level for tests and full reporting of outcomes
☒	☐	Estimates of effect sizes (e.g. Cohen's *d*, Pearson's *r*), indicating how they were calculated

Our web collection on statistics for biologists contains articles on many of the points above.

Software and code

Policy information about availability of computer code

Data collection	For data collection, we used various open-source Python 3.7.3 packages including but not limited to: jupyter==1.0.0,ipython==7.6.1,h5py==2.9.0, opencv-python==4.1.0, Pillow==6.1.0, pydicom==1.2.2. For a full list of the software dependencies used in our research as well as custom algorithms and software, see our Python package at: https://github.com/seyuboglu/weakly-supervised-petct.
Data analysis	For data analysis, we used various open-source Python 3.7.3 packages including but not limited to: jupyter==1.0.0,ipython==7.6.1,numpy==1.16.4, torch== 1.1.0, scipy==1.3.0, scikit-learn==0.21.2,nltk==3.4.4, and seaborn==0.9.0. For a full list of the software dependencies used in our research as well as custom algorithms and software, see our Python package at: https://github.com/seyuboglu/weakly-supervised-petct. We also performed some survival analyses with R version 3.6.1 and the rms package version 6.0.1.

For manuscripts utilizing custom algorithms or software that are central to the research but not yet described in published literature, software must be made available to editors/reviewers. We strongly encourage code deposition in a community repository (e.g. GitHub). See the Nature Research guidelines for submitting code & software for further information.

Data

Policy information about availability of data

All manuscripts must include a data availability statement. This statement should provide the following information, where applicable:

- Accession codes, unique identifiers, or web links for publicly available datasets
- A list of figures that have associated raw data
- A description of any restrictions on data availability

This research used data provided by STARR, “Stanford Medicine Research Data Repository,” a clinical data warehouse containing live Epic Clarity warehouse data from Stanford Health Care (SHC), the Stanford Childrens Hospital (SCH), the University Healthcare Alliance (UHA) and Packard Children’s Health Alliance (PCHA) clinics and other auxiliary data from 23 Hospital applications such as radiology PACS. Due to its containing protected health information, the full dataset used in this study will not be made publicly available except for the weakly-labeled FDG-PET/CT scans in the validation set and the manually-labeled FDG-PET/CT scans in the

test set. We will also release the parameters and raw outputs of all of our experiments (see our repository https://github.com/seyuboglu/weakly-supervised-petct). The raw metrics underlying Figures 2a, 3b, 3c, 4a, 4b, 4c, 4d and 5a are provided as Source Data files.

Field-specific reporting

Please select the one below that is the best fit for your research. If you are not sure, read the appropriate sections before making your selection.

- [x] Life sciences
- [ ] Behavioural & social sciences
- [ ] Ecological, evolutionary & environmental sciences

For a reference copy of the document with all sections, see nature.com/documents/nr-reporting-summary-flat.pdf

Life sciences study design

All studies must disclose on these points even when the disclosure is negative.

Sample size	8,251 FDG-PET/CT exams from 4,749 patients. This sample size corresponds to the number of PET/CT exams in STARR between 2003 and 2010 minus the the number of exams excluded according to the criteria described below. This dataset size proved sufficient for training abnormality detection models and evaluating their performance. However the performance of the framework would likely improve with increased sample size.
Data exclusions	We began with a raw dataset of 18,497 PET-CT exams across 11,251 patients, extracted from Stanford Hospital's archival records. We omitted all exams for which the PET and CT channels were fused (our framework operates on PET and CT channels separately, so it is not compatible with data in which the channels are fused into one series) . Of those that had separate PET and CT channels, we filtered out those exams with fewer than 50 slices (these were not full-body PET/CT scans, but instead only captured part of the body), those with a length disparity between the two modalities greater than 2 slices (in these exams either the PET or CT series had been truncated so the alignment between the two series was ambiguous), and those taken using head PET-CT protocols (these are not actually full-body PET-CT scans). The resulting dataset consisted of 8,251 exams. These exclusion criteria were defined prior to model development.
Replication	We trained all models with 5 different random seeds. All attempts at replication were successful. We report mean results across all random seeds throughout the manuscript.
Randomization	The patients in our dataset were randomly split into train (n=6,651 exams), validation (n=800 exams) and test (n=800 exams) sets. No covariates were controlled when splitting data.
Blinding	During model development, investigators only used data from the train and validation sets. We evaluated model performance on the test set only once and did not retrain the model or tune hyper-parameters after evaluating on the test set. Investigators were blinded to group allocation during data collection. Investigators were not blinded to group allocation during data analysis. The investigators needed to know which patients were in the train, validation and test sets in order to properly train, validate and test the models.

Reporting for specific materials, systems and methods

We require information from authors about some types of materials, experimental systems and methods used in many studies. Here, indicate whether each material, system or method listed is relevant to your study. If you are not sure if a list item applies to your research, read the appropriate section before selecting a response.

Materials & experimental systems

n/a	Involved in the study	
x		Antibodies
x		Eukaryotic cell lines
x		Palaeontology
x		Animals and other organisms
x		Human research participants
	x	Clinical data

Methods

n/a	Involved in the study	
x		ChIP-seq
x		Flow cytometry
x		MRI-based neuroimaging

Clinical data

Policy information about clinical studies

All manuscripts should comply with the ICMJEguidelines for publication of clinical research and a completedCONSORT checklist must be included with all submissions.

Clinical trial registration	N/A
Study protocol	There was no trial protocol as this research was not a clinical trial, but a retrospective study.
Data collection	The data in this study was acquired through STARR, "Stanford Medicine Research Data Repository," a clinical data warehouse containing live Epic Clarity warehouse data from Stanford Health Care (SHC), the Stanford Childrens Hospital (SCH), the University Healthcare Alliance (UHA) and Packard Children's Health Alliance (PCHA) clinics and other auxiliary data from Hospital applications such as radiology PACS. The data were accessed on October 11, 2018. The studies in the dataset were administered at Stanford hospital between 2003 and 2010. Dates of death for the cohort were retrieved using STARR, which integrates medical records at Stanford with the Social Security Death Index (SSDI). The date of acquisition for these dates was November 4, 2019.
Outcomes	We did not predefine any primary or secondary outcomes as this was not a clinical trial.
